# Comparing the yield and nutritional value of ensiled amaranth (*Amaranthus hypochondriacus*) cultivars with corn silage (*Zea mays*) in doublecropping condition

**DOI:** 10.1093/tas/txac158

**Published:** 2022-12-03

**Authors:** Sayed Ahmad Hosseini, Yousef Rouzbehan, H Fazaeli, Javad Rezaei

**Affiliations:** Department of Animal Science, Faculty of Agriculture, Tarbiat Modares University, P.O. Box 14115-336, Tehran, Iran; Department of Animal Science, Faculty of Agriculture, Tarbiat Modares University, P.O. Box 14115-336, Tehran, Iran; Animal Science Research Institute of Iran, Agricultural Research, Education and Extension Organization (AREEO), Karaj, Iran; Department of Animal Science, Faculty of Agriculture, Tarbiat Modares University, P.O. Box 14115-336, Tehran, Iran

**Keywords:** ensiled amaranth forage, chemical composition, in vitro disappearance of organic matter, degradability

## Abstract

Yield, chemical composition, and fermentation variables were compared for amaranth silages (AMS) from five cultivars (A5, A12, A14, A28, and Maria) and corn (*Zea mays*; CS). In vitro methane production, organic matter disappearance, microbial protein, ammonia-N concentration, volatile fatty acid levels, cellulolytic bacteria and protozoa populations, and in situ dry matter (DM) and crude protein (CP) degradability were evaluated. All crops were harvested when the plant was at the mid-milk line stage, then chopped, placed in sealed 5 L plastic bags and stored for 60 days. Data analysis was carried out using the PROC MIXED method of SAS with a randomized complete block design. The mean DM forage yield of CS was higher than the average DM yield of the amaranth cultivars (*P* < 0.001). In comparison with CS, the AMS had higher CP, lignin, ether extract, ash, calcium, phosphorus, magnesium, total phenolics and metabolizable protein (*P* < 0.001), but had lower DM, neutral detergent fiber, non-fiber carbohydrates, organic matter disappearance, lactic acid (*P* < 0.01) and in vitro methane production (*P* = 0.001). The AMS had higher (*P* < 0.01) pH, ammonia-N concentration, in vitro microbial protein, in situ digestible undegradable protein, and metabolizable protein compared to CS. Overall, in comparison to CS, the amaranths produced a silage of medium-quality.

## INTRODUCTION

Changes in climatic conditions leading to water scarcity in semi-arid areas may lead to a reduction in yield in conventional forage crops with subsequent shortages and price increases. The production of forage depends on soil and climate (Sotomayor-Ríos and Pitman, 2001), so there is a need to investigate alternative crops such as amaranth that are more tolerant of harsher conditions (Svirskis, 2009). Amaranth species are C4 dicotyledonous plants and therefore able to photosynthesize at high temperatures in conjunction with a higher water use efficiency than many C3 plants (Assad et al., 2017). These characteristics facilitate the introduction of amaranth as a crop to dry and marginal zones (Myers, 1996; Liu and Stützel, 2004). The yield of amaranth green mass exceeds by 20–30% the productivity of the traditional silage crop-corn, 13.2 t/ha DM *vs.* 12.0 t/ha DM for the Iowa farms, respectively (Mehrani et al., 2012; Langemeier and Purdy, 2019). The amaranth crop yields high levels of crude protein (CP) (up to 285 g/kg DM) and good DM digestibility (590–790 g/kg) with low levels (below toxic concentrations) of oxalic and nitric acids. This data suggests a good potential as ruminant forage (Abbasi et al., 2012). It has lower water requirement compared with corn which means that amaranth silage (AS) may be a better option where water availability is limited (Ofitserov, 2001).

Constant availability of animal feed is essential for the development of continuous livestock production. However, in many regions of the World, animal production is principally influenced by the variable and increasing tendency of feed price due to adverse climatic conditions, conflicts and wars in areas of production, competition from biofuel production, a variable exchange rate, and the fluctuating price of petroleum products used in the operation of farm machinery (Msangi et al., 2014). A possible solution to strengthen the self-sufficiency of concentrates and corn forage is by using a double cropping system in a single season, thus sustainably intensifying agricultural production. Double cropping is practiced in many areas of the world, where cereals such as wheat or barley are grown as the main crop followed by corn forage as a second crop (Kato, 2011; Nikkhah et al., 2011). Consequently, corn forage is harvested in late autumn (i.e., the temperature around 15–18 ^o^C), when there is inadequate heat and sunlight to allow for sufficient drying, and consequently a low DM silage (<250 g/kg fresh weight) is produced. In such climatic conditions, there is little information in the literature on the productive potential, nutritional characteristics, and fermentation patterns of *Amaranthus hypochondriacus* cultivars and their potential to replace corn forage. Therefore, this study describes amaranth and corn as the second crops in a double cropping system, using barley as the main crop followed by amaranth and corn to compare their suitability to be cultivated in this region (i.e., Malayer city, Hamedan Province, Iran) under these climactic conditions. The significant considerations for growers are to confirm yield and nutritional values by the selection of ideal cultivars under these conditions. Hence, this study was conducted to investigate how five amaranth (*A. hypochondriacus*) cultivars when both had low DM content (<250 g DM/kg fresh weight) responded to climate in terms of biomass production, and accumulation of nutrients when compared to corn forage.

## MATERIALS AND METHODS

All aspects of animal care in this experiment were taken from The Guide for the Care and Use of Agricultural Animals in Research and Teaching (FASS, 2020). The protocols of the study (#9530381003, 2017) had the approval of the Animal Science Committee of Tarbiat Modares University.

### Forage Preparation and Sampling Methods

The Seed and Plant Research Improvement Institute (Karaj) supplied corn seeds (*Zea mays* hybrid variety SC 704). The amaranth cultivars’ seeds (A5, A12, A14, A28, and Maria) were supplied by Sven-Erik Jacobsen (Quinoa Quality, Regstrup, Denmark) and had been selected for their performance from the PROTEIN2FOOD project. The experimental location was near Malayer city (Hamadan Province) at 1751 m above sea level (Taherimoghaddam et al., 2018) with a mean yearly rainfall of 343 mm (2017) and a mean temperature of 12.4 °C (Hamadan Regional Water Authority, 2022) in soft loam-clay soil (Sand: 20%, Clay: 30%, Silt: 50%) (Naderi-Mahdei and Bahrami, 2014). There were four 25 m^2^ replicate plots per treatment set out as a randomized complete block design. The amaranths were sown into coir beds in culture trays (at a seeding rate of approx. 0.5 kg/ha) then transplanted when 10 cm high (industry standard). The corn was direct precision drilled (Tarashkadeh Co., Karaj, Iran). There was a 50 cm row spacing for all plants in the field. Nitrogen fertilizer in the form of urea had been applied to the plots at a rate of 53 kg N/ha. Weeds were controlled by row cultivation until the plants became established. When in the beds, the amaranth was irrigated daily but the corn and the amaranth transplants in the field were irrigated every other day with a drip irrigation system (20 cm hole spacing, bar type). The mean total irrigation applied to corn and amaranth cultivars were 350 and 250 mm (equivalent to 3,500 and 2,500 m^3^/ha), respectively as it has been shown that amaranth needs less water than corn to achieve the same biomass (260 *vs.* 370 g/g) (Ofitserov, 2001) and Shadi et al. (2020) recorded no detrimental effects on amaranth growth at this level. The crops were harvested, manually, in 85 days (October 25, 2017) (Nikkhah et al., 2011; Abbasi et al., 2018) at the mid-milk line stage (Lancashire et al., 1991) and then left to wilt for 30 hours. The DM values of corn and amaranths, Maria, A5, A12, A14, and A28, at harvest were 184, 163, 130, 158, 127, and 117 g/kg wet weight, respectively. The DM after wilting of the crops are shown in Table 2. The crops were chopped, by knife, into approximately 2 cm lengths and packed tightly into 10 L plastic bags, without a microbial inoculant, vacuum sealed and left for 60 days (Shadi et al., 2020). Fresh forage and silage samples were kept for analysis.

**Table 2.** Dry matter (g/kg as-fed) and chemical composition (g/kg DM) of the fresh forages and silages from five amaranth varieties (Maria, A5, A12, A14, A28) and corn.

Item^1^	Fresh forage									Ensiled forage								
	Amaranth varieties							*P*-value		Amaranth varieties							*P*-value	
	Corn	Maria	A5	A12	A14	A28	SEM^2^	CA^3^	AM^4^	Corn	Maria	A5	A12	A14	A28	SEM^2^	CA^3^	AM^4^
DM	202	185^a^	141^cd^	174^b^	149^c^	133^d^	1.85	0.003	0.01	217	187^a^	152^b^	177^a^	155^b^	152^b^	2.92	<0.001	0.004
CP	77	160^b^	158^b^	148^c^	156^b^	170^a^	1.67	<0.001	0.008	77	155^b^	144^c^	141^c^	142^c^	163^a^	3.09	<0.001	0.002
NDFom	493	370^a^	351^c^	378^a^	357^b^	339^d^	2.55	0.003	0.009	482	353^a^	335^b^	355^a^	341^b^	317^c^	3.22	<0.001	0.006
ADFom	308	279^b^	252^c^	290^a^	273^b^	244^c^	2.24	0.01	0.005	308	271^a^	243^bc^	281^a^	262^ab^	234^c^	2.88	0.003	0.005
ADL	48.3	63.4^a^	60.4^ab^	58.9^b^	65.0^a^	67.2^a^	1.96	0.003	0.007	47.6	62.4^ab^	60.0^b^	56.5^c^	64.2^a^	65.2^a^	1.91	<0.001	0.002
WSC	120	57^a^	55^ab^	53^b^	52^b^	50^b^	1.57	<0.001	0.03	11.0	12.2^b^	13.5^b^	14.0^a^	12.0^b^	11.2^c^	1.05	0.001	<0.001
EE	26.4	43.9^a^	39.2^b^	36.1^b^	38.3^b^	33.0^b^	0.54	<0.001	0.006	26.0	56.2^a^	52.9^ab^	48.0^b^	48.1^b^	43.0^c^	1.18	<0.001	0.008
Ash	72	177^c^	197^b^	198^b^	204^b^	222^a^	2.63	<0.001	0.005	71	197^d^	227^b^	207^c^	223^b^	239^a^	2.89	<0.001	0.004
NFC	332	249^b^	255^a^	240^c^	245^bc^	236^c^	3.05	0.002	0.004	344	239^b^	251^a^	249^a^	246^a^	238^b^	3.42	<0.001	0.002
Ca	3.2	13.3	15.5	14.0	14.7	15.1	1.01	<0.001	0.28	3.3	13.9	15.9	14.4	15.0	15.4	0.92	<0.001	0.38
P	2.4	5.3	5.5	4.9	5.1	5.2	0.59	0.001	0.33	2.5	5.3	5.6	4.9	5.2	5.2	0.45	<0.001	0.15
Mg	2.1	5.4	4.5	4.8	4.7	4.4	0.24	<0.001	0.25	2.3	5.5^a^	4.6	4.8	4.9	4.4	0.46	<0.001	0.11

^1^DM = dry matter; CP = crude protein; NDFom = ash-free NDF; ADFom = ash-free ADF; ADL = acid detergent lignin; EE = ether extract; WSC = water-soluble carbohydrates; EE = ether extract; NFC = non-fiber carbohydrates (on DM basis) calculated as: 1000 – (NDFom g/kg + CP g/kg + EE g/kg + ash g/kg); ^2^SEM = standard error of the means; ^3^CA = comparing corn *vs.* average value of amaranths; ^4^AM = comparing among amaranth varieties; ^a, b, c, d, e^ among amaranths = means in the same row with different superscripts differ (*P* < 0.05).

### Chemical Analyses

Samples of silage were weighed and dried at 60 °C for 48 h to determine DM percentage then ground to pass through a 1-mm sieve. Total nitrogen (N) was determined using method 990.03 of AOAC (2012) with CP being calculated as 6.25 times the total N concentration. Total ash content was measured using the method from AOAC (2012) as were calcium, magnesium and phosphorus concentrations. Ether extract (EE) was determined using AOAC (2012) method 2003.05. Neutral detergent fiber (NDF) levels were determined using alpha amylase, without sodium sulfite, and expressed exclusive residual ash (NDFom; Van Soest et al., 1991). Ash-free acid detergent fiber (ADFom) was determined using AOAC (2012) method (973.18) and acid detergent lignin (ADL) using AOAC (2012) method (973.18). Water-soluble carbohydrates (WSC) concentration was measured by the anthrone reaction assay (MAFF, 1986). The nonfiber carbohydrates (NFC) content (on a DM basis) was calculated as 1000 – (NDFom g/kg + CP g/kg + EE g/kg + ash g/kg) (NRC, 2001). Nitrate concentration was measured using colorimetry (Singh, 1988). The oxalic acid concentration was measured by spectrophotometry using the method of Savage et al. (2000). The Folin–Ciocalteau method (Makkar, 2000) was used to determine total phenolics. Non-tannin phenolics were measured by the Folin–Ciocalteau reaction after the absorption of tannins to insoluble polyvinylpyrrolidone. (Makkar, 2000). Total tannin levels were obtained by subtracting non-tannin phenolics from total phenolics with the data expressed as tannic acid (Merck GmbH, Germany).

For measuring silage pH, 50 g of fresh silage was blended with 125 mL of distilled water and allowed to stand at room temperature for 1 h (Faithfull, 2002). After decanting the silage extract into a small beaker, the pH was measured using a digital pH meter (Sartorius PT-10; Germany). Two milliliters of juice from the silages were pipetted into centrifuge tubes containing 0.2 mL of acid (25% meta-phosphoric acid and 2-ethyl butyric acid 2 g/L as the internal standard), then centrifuged at 10,000 *× g* for 10 min at 4 °C (Galyean, 2010). Volatile fatty acids in the supernatant were quantified using gas chromatography (UNICAM 4600; SB Analytical, Cambridge, UK) with a flame ionization detector (FID; 250 °C), split-injection port (1.0 μL injection), capillary column (Agilent J & W HP-FFAP, 10 m by 0.535 mm by 1.00 µm, 19095 F-121; Agilent, CA), and helium as the carrier gas (column head pressure of 10 psi). To determine NH_3_-N, an extract was obtained by squeezing the silage material, filtered using Whatman 54 filter paper, then a 9 mL of aliquot was taken, mixed with 1 mL of 7.2 N H_2_SO_4_, and stored at −20 °C. After thawing, the silage extracts were analyzed for NH_3_-N using a phenol-hypochlorite assay (Galyean, 2010).

### In vitro Ruminal Gas Production and Estimated Variables

A 24-h in vitro gas production (GP) was conducted to determine the silage fermentation properties according to the method of Menke et al. (1979). Rumen fluid was used from 3 fistulated Shall sheep that had been fed twice daily on a diet containing (g/kg DM) 400 alfalfa hay, 150 corn silage (CS), 100 amaranth silages, 150 rolled barley, 140 wheat bran, 50 soybean meal, and 10 vitamin-mineral premix. Rumen fluid was collected before the sheep’s morning feed. One hundred and ninety-eight syringes (six treatments × four blocks × two individual samples per block × two syringes per sample × two runs, with three blanks in each run) were used. The samples (200 mg DM) were incubated in 30-mL of diluted rumen liquor (10 mL of the rumen fluid + 20 mL buffer) under a CO_2_ atmosphere at 39 °C. The gas volume was measured from the change in the position of the syringe piston after 24-h. The *in vitro* OMD and metabolizable energy (ME) were calculated using the following equations:


$$\begin{array}{ll} OMD\left(g/kg\right) & =148\cdot 8+(8\cdot 893\times GP_{24})\\ & +(0\cdot 448\times CP)+(0\cdot 651\times XA) \end{array}$$

$$\begin{array}{ll} ME\left(MJ/kg\;DM\right) & =2\cdot 20+(0\cdot 1357\times GP_{24})\\ & +(0\cdot 0057\times CP)+(0\cdot 00002859\times CP^{2}) \end{array}$$

In the above equations, GP_24_ is net gas produced after 24 h (mL/200 mg DM), CP is crude protein (g/kg DM), XA is ash (g/kg DM).

Eight syringe contents per treatment from each run (four blocks × two individual samples × one syringe per sample) after soluble product removal (Van Soest et al., 1991) were used to determine truly degraded substrate (TDS) levels (Blümmel et al., 1997). The partitioning factor after 24 h (PF_24_) was calculated from Blümmel et al. (1997)$PF_{24}\left(mg/mL\right)=TDS\left(mg\right)/GP_{24}\left(mL\right)$, where GP_24_ is 24-h gas production. Microbial CP (MCP, mg/g DM) was estimated from the equation $TDS\left(mg\right)-\left(mLGP_{24}\times 2.2mg/mL\right)$, 2.2 mg/mL being a stoichiometric factor expressing mg of carbon, hydrogen, and oxygen required for the volatile fatty acids-gas complex production needed for 1 mL of gas produced at 24 h”. Eight syringes per treatment per run (four blocks × two samples) were used to determine *in vitro* pH, NH_3_-N, VFA and numbers of cellulolytic bacteria, and protozoa. Ammonia-N was measured from a strained sample of 2.5 mL by the method of Galyean (2010). The VFA levels were measured in 2mL of supernatant using gas chromatography by the method of Galyean (2010). Total protozoa numbers and subfamily counts were enumerated using the method of Dehority (2003). The cellulolytic bacteria population was determined using Hungate tubes containing media according to Bryant (1972). After 21 days the Most Probable Number method of Dehority (2003) was used to ascertain bacteria numbers. Gas production (GP) kinetics were evaluated from a 96-h in vitro experiment with gas being recorded at 2, 4, 6, 8, 12, 24, 48, 72, and 96 h of the incubation. Kinetic variables were predicted according to Carro et al. (1999):- “$y\;=\;B\;\left(1\;-\;e^{-\;c(t-L)}\right)$. In this model, y, B, c, and L are the gas volume detected at time t, asymptotic value of produced gas (mL/200 mg diet DM), first-order fractional rate constant of produced gas (/h), and lag time (h), respectively”. The in vitro methane produced from each sample was measured after 24-h incubation (Menke et al., 1979). After the total gas volume had been recorded, four mL of 10 M NaOH was introduced into the syringe to absorb CO_2_ leaving the remainder to be measured as CH_4_ (Demeyer et al., 1988).

### In situ Degradability of DM and CP

The silage DM and CP degradability was determined by the in situ method (AFRC, 1992) from bags suspended in the rumens of four fistulated Shall sheep. The sheep were fed the diet (as above) for 2 weeks to allow for adaptation. The bags were 10 × 21 cm having a 45 µm pore size (Bucksburn, Aberdeen, UK). Dried silage samples were ground to pass through a 4 mm sieve using a Cyclotec TM 1093 Sample Mill (Foss Company, Denmark). The bags each contained 5 g DM and were incubated for 2, 4, 8, 12, 24, 48, 72, and 96 h. One bag per sample was used for each time in each sheep. When the bags were removed, they were cold water washed (Hoover OPHS 612; Hoover, London, UK) for 1 h, then dried to a constant weight at 55 ^o^C. The degradability value at time zero was determined from 3 bags per sample washed and dried as above. Bags and contents were weighed to estimate degraded DM and residues were analyzed for CP according to AOAC (2012) method (942.05). The degradation kinetics parameters of DM and CP were determined by the method of Ørskov and McDonald (1979): An exponential curve as $Y\;=\;a\;+\;b\;\left(1\;-\;e^{-ct}\right)$ fitted to the experimental data by iterative regression analysis. The effective degradability (ED) of DM and CP was then estimated as ED(%)=a+[(b+c)/(c+k)]. In these equations, ‘Y’ is disappearance from the bag, ‘a’ is the soluble and very rapidly degradable fraction, ‘b’ is the insoluble but potentially degradable fraction degrading at a constant fractional rate (c) per unit time (t), ‘e’ is the base of natural logarithms, ED is the effective degradability, and ‘k’ refers to the fractional outflow rate of small particles from the rumen. An assumed value for ‘k’ was 0.02 fraction/h (Ørskov and McDonald, 1979). Effective RDP (ERDP) was calculated from the equation recommended by AFRC (1992) as:


$$\begin{array}{l} ERDP=CP\times \left[0.8a+bc/\left(c+r\right)\right] \end{array}$$

where a, b, and c are the fitted parameters derived from in situ determination of feed degradability, 0.8 is the efficiency of capture of the nitrogen of the readily degradable fraction, and r is the outflow rate (0.02 fraction/h). The DUP was calculated as 0.9 (UDP - 6.25 ADIN), where UDP is dietary undegraded protein, and ADIN is acid detergent insoluble nitrogen (which is undegradable and indigestible in the rumen and abomasum). The proportion of the microbial CP (*i.e.,* ERDP) present as true protein is 0.75 (amino acids) and its true digestibility is 0.85, therefore, the MP is calculated as: MP (g/kg DM) = [ERDP × 0.75 × 0.85] + UDP

### Statistical Analysis

The SAS, PROC MIXED program (SAS Inst. Inc., Cary, NC) was used for the analysis of data from yield and chemical composition of the forage and silage treatments, as well as fermentation quality of the silages, using a randomized complete block design with treatment being a fixed effect and random effect of block. The model was Y_ijk_ = μ + T_i_ + B_j_ + e_ij_ + e_ijk_, where Y_ijk_ is the observation, μ is the general mean, T_i_ is the treatment effect, B_j_ is the block effect and e_ij_ and e_ijk_ are the experimental and sampling errors respectively. Gas production data of the silage treatments was analyzed using a randomized complete block design as described above, including the random effect of run. Moreover, the SAS, PROC MIXED, program was used for the in situ study data analysis. The model included the fixed effect of treatment and the random effects of animal and block. The PROC UNIVARIATE was used to test the residual normality of the data.

Least squares means (five means) of the amaranths were separated using the DIFF option. Moreover, the single contrast test was used to compare corn (as a treatment) *vs.* average amaranth value (the mean of five amaranths). The *P*-values ≤ 0.05 were accepted to define statistical significance.

## RESULTS

### Yield, Chemical Analyses, and Antinutritional Factors

In Table 1, results of fresh forage illustrated that dry forage yield/ha of the corn was higher (*P* = 0.002) than the mean amaranth yield (6.5 *vs.* 5.4). Amaranth forages had lower (*P* < 0.01) overall mean levels of DM (156 *vs.* 202), NDFom (359 *vs.* 493), and NFC (245 *vs.* 332), but higher (*P* < 0.01) ADL (63 *vs.* 48), CP (158.4 *vs.* 77), EE (39 *vs.* 26), ash (199 *vs.* 72), calcium (14.5 *vs.* 3.2), phosphorus (5.2 *vs.* 2.4), and magnesium (4.7 *vs.* 2.1) levels (Table 2). Among amaranth cultivars, Maria had the highest (*P* = 0.01) DM value. The A28 forage had the highest (*P* = 0.008) CP value, followed by Maria, A5, A14, with A12 being the lowest. The NDFom values for Maria and A12 were higher (*P* = 0.003) than the rest. In comparison to corn, amaranth fresh forages had lower (*P* < 0.001) overall mean levels of WSC (53 *vs.* 120). Among amaranths, Maria had a higher (*P* = 0.03) WSC value in comparison with A12, A14, and A28. Compared to amaranth forage, corn forage had lower (*P* < 0.001) EE (26.4 *vs.* 38.1). Maria forage showed the highest (*P* = 0.006) EE concentration among amaranth cultivars. The ash content of corn forage was lower (*P* < 0.001) than the mean value of amaranths (72 *vs.* 200). Among amaranth forages, A28 had higher ash level (*P* = 0.005) than the rest. The highest and lowest (*P* = 0.004) NFC values for amaranth forages were in A5 and A28 (255 *vs.* 236). In comparison to corn forage, amaranth cultivars had higher (*P* < 0.01) calcium (3.2 *vs.* 14.5), phosphorus (2.4 *vs.* 5.2), and magnesium (2.1 *vs.* 4.7) concentrations. There were no differences in calcium, phosphorus and magnesium concentrations among the amaranth forages. In Table 3, results of fresh forages show that the corn forage showed lower levels of nitrate (0.9 *vs.* 2.5), total phenolics (3.6 *vs.* 8.9), and total tannins (2.2 *vs.* 4.6), but higher oxalate (8.2 *vs.* 6.6) than the mean amaranth values (*P* < 0.01). In terms of amaranth forage, Maria had the highest (*P* < 0.001) nitrate and total phenolics values.

**Table 1.** Yields of the fresh forages and silages from corn and five amaranth varieties.

Yield	Amaranth varieties							*P*-value	
	Corn	Maria	A5	A12	A14	A28	SEM^2^	CA^3^	AM^4^
Fresh forage, t/ha	30.0	32.5^c^	29.2^f^	35.8^a^	34.3^b^	31.6^d^	2.19	0.001	0.002
Dry forage, t/ha	6.5	6.1^a^	4.4^c^	6.4^a^	5.3^b^	4.8^b^	0.47	0.002	0.002
Silage									
Dry silage, t/ha	6.50	6.10^b^	4.43^e^	6.35^a^	5.33^c^	4.80^d^	0.47	<0.001	0.002
CP, t of DM/ha	0.50	0.94^a^	0.64^e^	0.90^b^	0.76^d^	0.78^c^	0.032	0.005	0.007
MP,S t of DM/ha	0.28	0.60^a^	0.38^e^	0.57^b^	0.46^d^	0.46^c^	0.013	0.01	0.008
OM, t of DM/ha	5.10	4.94^b^	3.76^e^	5.23^a^	4.50^c^	4.06^d^	0.30	<0.001	0.003
OMD, t of DM/ha	3.28	2.57^c^	2.25^e^	3.22^a^	2.36^d^	2.16^f^	0.57	0.003	<0.001

^1^DM = dry matter; CP = crude protein estimated from total nitrogen content × 6.25; MP = metabolizable protein; OM = organic matter; OMD = organic matter disappearance calculated as 148.8 + (8.893 × GP_24_) + (0.448 × CP) + (0.651 × XA), (Menke et al., 1979). ^2^SEM = standard error of the means; ^3^CA = comparing corn *vs.* average value of amaranths; ^4^AM = comparing among amaranth varieties; ^a,b, c, d, e, f^ among amaranths = means in the same row with different superscripts differ (*P* < 0.05).

**Table 3.** Anti-nutritional factors content (g/kg DM) of the fresh forages and silages of five amaranth varieties (Maria, A5, A12, A14, A28) and corn

Item	Fresh forage									Ensiled forage								
	Amaranth varieties							*P*-value		Amaranth varieties							*P*-value	
	Corn	Maria	A5	A12	A14	A28	SEM^3^	CA^4^	AM^5^	Corn	Maria	A5	A12	A14	A28	SEM^3^	CA^4^	AM^5^
Nitrate	0.9	3.2^a^	2.9^b^	2.6^bc^	2.2^c^	1.7^d^	0.24	<0.001	0.001	0.8	1.9^a^	1.6^b^	1.7^a^	1.7^a^	1.2^c^	0.19	<0.001	0.001
Oxalate	8.2	4.4^c^	7.3^b^	4.7^c^	9.3^a^	7.4^b^	0.31	0.004	<0.001	7.4	4.1^c^	7.0^b^	4.5^c^	8.9^a^	7.1^b^	0.42	0.006	<0.001
TP^1^	3.6	10.6^a^	8.9^b^	9.5^b^	7.7^c^	7.7^c^	0.31	<0.001	0.006	2.2	7.6^a^	6.4^b^	6.1^c^	4.8^cd^	4.1^d^	0.14	<0.001	<0.001
TT^2^	2.2	6.7^a^	4.5^b^	4.7^b^	3.4^c^	3.7^c^	0.19	<0.001	<0.001	1.2	4.2^a^	3.2^b^	2.8^b^	1.8^c^	1.7^c^	0.15	<0.001	<0.001

^1^TP = total phenolics; TT^2^ = total tannins; ^3^SEM = standard error of the means; ^4^CA = comparing corn *vs.* average value of amaranths; ^5^AM = comparing among amaranth varieties; ^a, b, c, d^ among amaranths = means in the same row with different superscripts differ (*P* < 0.05).

According to Table 1, CS had higher (*P* < 0.01) yields of DM (6.5 *vs.* 5.4), OM (5.1 *vs.* 4.5), and OMD (3.28 *vs.* 2.5), but lower (*P* < 0.01) CP (0.5 *vs.* 0.8) and MP (0.28 vs. 0.5) yields in comparison with the mean values of AMS. Silage of A12 cultivar had the highest (*P* < 0.001) DM yield/ha, and A5 had the lowest among AMS (6.35 *vs.* 4.43). In terms of OM and OMD yields, A12 silage had the highest (*P* < 0.01) followed by Maria, A14, A28, and A5 having the least (*P* < 0.01). The A12 had the highest silage DM yield (6.35) whereas Maria had the highest CP yield (0.94) among all the AMS (*P* < 0.01). In comparison to CS, amaranth silages had lower (*P* < 0.001) overall mean levels of DM (165 *vs.* 217), NDFom (340 *vs.* 482), and NFC (245 *vs.* 344), but higher (*P* < 0.01) CP (149 *vs.* 77), ADL (61.7 *vs.* 47.6), WSC (12.6 *vs.* 11), EE (49.6 *vs.* 26.0), ash (219 *vs.* 71), calcium (14.9 *vs.* 3.3), phosphorus (5.2 *vs.* 2.5), and magnesium (4.8 *vs.* 2.3) levels (Table 2). Among the AMS, A12 and Maria had higher (*P* < 0.01) NDFom values than A14, A28 and A5. The A12 silage had the lowest (*P* = 0.002) ADL value. In amaranth silages, A28 had the lowest (*P* < 0.001) WSC value and A12 the highest (*P* < 0.01) (11.2 *vs.* 14.0). Maria silage showed the highest (*P* < 0.001) EE concentration and A28 had lowest ash level (*P* = 0.004) in comparison with the other cultivars. Among amaranth silages, A12 and A5 had the highest (*P* = 0.002) NFC values, followed by A14, Maria, and A28. There were no differences in calcium, phosphorus and magnesium concentrations among the ensiled amaranth. Table 3 shows the results of silages which illustrated that corn silage showed lower (*P* < 0.001) levels of nitrate (0.83 *vs.* 1.6), total phenolics (2.2 *vs.* 5.8), and total tannins (1.2 *vs.* 2.7), but higher (*P* = 0.006) oxalate (7.4 *vs.* 6.3) than the mean of AMS values. Among AMS, Maria, A12, and A14 had higher (*P* = 0.001) nitrate values in comparison to A5 and A28. Moreover, A14 had the highest (*P* < 0.001) oxalate content and A12 had the lowest.

### Silage Fermentation Characteristics

The mean pH values and ammonia-N levels in AMS were higher (*P* < 0.001) than in CS, but CS had higher values (*P* < 0.001) of acetate and lactate (Table 4). Propionic acid levels showed no difference between CS and AMS (*P* = 0.25) and among ensiled amaranth (*P* = 0.08). Among AMS, Maria had the lowest (*P* = 0.002) butyric acid level and A28 the highest. In terms of AMS, A28 had higher (*P* = 0.007) pH and ammonia-N values but a lower (*P* < 0.001) lactic acid level. Maria and A12 had the highest acetic acid levels followed by A5 and A14 with A28 having the lowest (*P* < 0.001).

**Table 4.** Silage fermentation characteristics of five amaranth varieties (Maria, A5, A12, A14, A28) and corn.

Item	Amaranth varieties							*P*-value	
	Corn	Maria	A5	A12	A14	A28	SEM^1^	CA^2^	AM^3^
pH	3.90	4.28^d^	4.78^b^	4.36^c^	4.60^d^	4.96^a^	0.03	<0.001	0.007
Ammonia-N, g/kg total N	42.3	53.5^c^	64.0^b^	57.0^c^	56.5^c^	66.6^a^	1.86	<0.001	0.002
Lactic acid, g/kg DM	70.2	62.1^a^	55.8^b^	60.1^a^	60.0^a^	51.0^c^	1.94	<0.001	<0.001
Volatile fatty acids									
Acetic acid, g/kg DM	23.3	19.4^a^	17.1^b^	18.9^a^	16.7^b^	15.9^c^	0.86	<0.001	<0.001
Propionic acid, g/kg DM	2.8	2.1	2.5	2.6	3.1	3.2	0.31	0.25	0.08
Butyric acid, g/kg DM	0.68	0.64^c^	0.92^b^	0.67^c^	0.90^b^	1.06^a^	0.09	0.05	0.002

^1^SEM = standard error of the means; ^2^CA = comparing corn *vs.* average value of amaranths; ^3^AM = comparing among amaranth varieties; ^a^, ^b, c, d^ among amaranths = means in the same row with different superscripts differ (*P* < 0.05).

### In vitro Gas Production and Estimated Variables

There was no difference between the in vitro ruminal pH of CS and that of the mean AMS value (Table 5) and neither was there among the AMS in vitro pHs. The ammonia-N value in CS was lower (*P* < 0.001) than the AMS mean value. The in vitro total VFA level, the molar proportion of acetate, and the acetate-to-propionate ratio were higher (*P* < 0.01) in CS than the AMS mean value, but CS had a lower (*P* < 0.001) in vitro butyric acid level. There was no difference in the in vitro isovaleric acid molar proportion between CS and the mean value from the amaranths. There were no differences in the in vitro ruminal proportions of acetic, propionic, butyric and isovaleric acids, or acetate to-propionate ratio among the AMS, but in vitro total VFA levels in A5, A14, and A28 were lower (*P* = 0.02).

**Table 5.** In vitro ruminal fermentation characteristics, protozoa count (× 10^5^/mL digesta) and cellulolytic bacteria enumeration (log_10_/mL digesta) of five amaranth varieties (Maria, A5, A12, A14, A28) and corn.

Item	Amaranth							*P*-value	
	Corn	Maria	A5	A12	A14	A28	SEM^1^	CA^2^	AM^3^
pH	6.70	6.67	6.71	6.76	6.80	6.82	0.13	0.19	0.11
Ammonia-N, mg/dL	10.7	22.1^c^	22.8^b^	23.7^ab^	24.3^a^	24.4^a^	0.39	<0.001	0.002
Total VFA^4,^ mmol/L	60.9	58.1^a^	55.0^b^	57.6^a^	55.5^b^	55.2^b^	1.36	<0.001	0.02
VFA (mol/100 mol)									
Acetic acid	70.80	66.12	66.9	66.1	65.9	65.9	0.85	0.005	0.38
Propionic acid	21.0	22.9	22.6	22.5	221.0	22.6	0.32	0.45	0.19
Butyric acid	4.55	6.36	6.48	7.21	7.46	7.60	0.83	<0.001	0.12
Isovaleric acid	3.67	4.59	4.00	4.15	4.65	3.93	0.73	0.56	0.31
A:P^5^	3.37	2.90	2.91	2.93	2.99	3.03	0.25	0.009	0.37
Total protozoa	7.45	6.07	6.20	6.14	6.17	6.09	0.11	0.022	0.26
*Isotrichidae*	1.83	1.55	1.61	1.70	1.70	1.60	0.068	0.22	0.21
*Entodiniinae*	3.43	2.62	2.85	2.63	2.54	2.60	0.090	<0.001	0.18
*Diplodiniinae*	1.46	1.09	1.01	1.06	1.10	1.13	0.057	0.040	0.54
*Ophryoscolecina*	0.73	0.71	0.73	0.75	0.83	0.76	0.063	0.35	0.25
Cellulolytic bacteria	8.23	8.01	8.01	8.08	7.96	7.90	0.10	0.005	0.66

^1^SEM = standard error of the means; ^2^CA = comparing corn *vs.* average value of amaranths; ^3^AM = comparing among amaranth varieties; ^4^VFA = volatile fatty acids; ^5^A:P = Acetic acid: Propionic acid ratio; ^a, b, c^ among amaranths = means in the same row with different superscripts differ (*P* < 0.05).

The in vitro ruminal total protozoa population of CS was higher (*P* = 0.022) than the mean value from AMS (Table 5). The cellulolytic bacteria numbers in CS were higher (*P* = 0.005) than the mean value from AMS.

The in vitro 24-h CS incubation had higher (*P* < 0.001) GP_24_, OMD, ME, TDS, and CH_4_, but lower PF_24_ and MCP (Table 6) in comparison with the mean value from the AMS. Concerning the CS 96-h incubation, the ‘b’ value was higher and the ‘c’ value lower (*P* < 0.001) than the mean AMS value. The A12 cultivar had the highest (*P* < 0.01) in vitro GP_24_, OMD, ME, and TDS and the lowest (*P* = 0.001) PF_24_ of the AMS. The A28 cultivar had lower in vitro CH_4_ production, but there was no difference among other AMS.

**Table 6.** In vitro ruminal gas production and estimated variables, truly degraded substrate and microbial protein of the corn and five amaranth varieties silages.

Item^1^	Amaranth varieties							*P*-value	
	Corn	Maria	A5	A12	A14	A28	SEM^2^	CA^3^	AM^4^
24-h incubation									
GP, mL/200 mg DM	46.5	19.5^c^	27.3^b^	30.5^a^	18.7^c^	17.5^c^	0.81	<0.001	<0.001
OMD, g/kg	644	520^c^	598^b^	617^a^	524^c^	531^c^	2.89	<0.001	<0.001
ME, MJ/kg DM	9.13	6.41^b^	7.32^a^	7.72^a^	6.13^b^	6.27^b^	0.12	<0.001	0.004
TDS, g/kg DM	682	582^a^	580^a^	620^a^	487^b^	451^c^	3.43	<0.001	<0.001
PF_24,_ g TDS/mL GP per g DM	2.93	5.97^a^	4.24^c^	4.06^d^	5.20^b^	5.15^b^	0.16	<0.001	0.001
MCP, g/kg DM	133	368^a^	279^b^	285^b^	281^b^	259^c^	3.53	<0.001	<0.001
CH_4,_ % of total GP	22.8	20.5^a^	19.5^a^	20.5^a^	20.0^a^	18.0^b^	0.66	0.001	0.004
96-h incubation									
b, mL/200 mg DM	60.78	42.53^b^	44.58^ab^	46.81^a^	41.80^b^	38.34^c^	0.95	<0.001	<0.001
Lag time, h	0.059	0.066	0.067	0.063	0.068	0.067	0.010	0.34	0.70
c, /h	0.037	0.046^b^	0.044^b^	0.041^c^	0.049^ab^	0.052^a^	0.002	<0.001	<0.001

^1^GP24 = in vitro ruminal gas production at 24 h; OMD = estimated organic matter disappearance; ME = metabolizable energy; TDS = truly degraded substrate; PF = partitioning factor at 24 h of incubation; MCP = microbial crude protein; B = the asymptotic value of gas production; C = constant rate of gas production; ^2^SEM = standard error of the means; ^3^CA = comparing corn *vs.* average value of amaranths; ^4^AM = comparing among amaranths; ^a, b, c^ among amaranths = means in the same row with different superscripts differ (P < 0.05).

In regard to the AMS in vitro 96-h incubation, the lowest ‘b’ value (*P* < 0.001) was recorded in A14 with A5 having the highest value. The highest ‘c’ value (*P* < 0.001) was recorded in A28 with the lowest value in A12.

### In situ Degradability of DM and CP

The CS had more (*P* < 0.01) insoluble-degradable DM, ‘c’ and ED of DM, but less (*P* < 0.001) soluble DM compared to the AMS mean values (Table 7). Among the AMS, A12 had a higher (*P* < 0.001) ‘b’ fraction and ED of DM whereas A5 had the lowest ‘b’ and ED of DM. The highest and lowest (*P* < 0.001) values of ‘cʹ among AMS were recorded in A28 and A12, respectively.

**Table 7.** In situ degradability of dry matter and crude protein of the silages of corn and five amaranth varieties silages.

Item^1^		Amaranth varieties						*P*-value	
	Corn	Maria	A5	A12	A14	A28	SEM^2^	CA^3^	AM^4^
DM degradation									
a, g/kg DM	344	351^b^	370^a^	368^a^	371^a^	357^b^	2.95	<0.001	<0.001
b, g/kg DM	479	439^bc^	427^c^	464^a^	444^b^	428^c^	2.76	<0.001	<0.001
c, /h	0.051	0.043^b^	0.044^b^	0.041^b^	0.043^b^	0.056^a^	0.004	<0.001	<0.001
ED, g/kg	607	554^c^	542^d^	586^a^	574^b^	578^b^	3.15	<0.001	<0.001
CP degradation									
a, g/kg CP	488	460^b^	476^a^	450^c^	466^b^	468^b^	2.38	<0.001	<0.001
b, g/kg CP	412	498^a^	460^cd^	468^c^	455^d^	482^b^	3.88	<0.001	<0.001
ADICP, g/kg CP	100	42^c^	64^b^	82^a^	79^a^	50^c^	1.23	<0.001	<0.001
c, /h	0.027	0.025^c^	0.031^b^	0.018^d^	0.027^c^	0.045^a^	0.003	<0.001	<0.001
ERDP, g/kg DM	48.0	99.9^b^	95.1^b^	82.0^d^	90.1^c^	115.0^a^	2.22	<0.001	<0.001
DUP, g/kg DM	12.09	30.87^a^	23.38^bc^	31.26^a^	24.74^b^	21.74^c^	1.75	<0.001	<0.001
MP, g/kg DM	42.83	94.83^a^	84.24^b^	83.75^b^	82.38^b^	95.62^a^	1.62	<0.001	<0.001

^1^a = soluble and very rapidly degradable fraction; b = insoluble but potentially fermentable fraction; c = fractional degradation rate of b; ED = effective degradability calculated for an outflow rate of 0.02/h; ADICP = Acid detergent insoluble crude protein; ERDP = effective rumen degradable protein; DUP = digestible undegradable protein; MP = metabolizable protein. ^2^SEM = standard error of the means; ^3^CA = comparing corn *vs.* average value of amaranths; ^4^AM = comparing among amaranths; ^a, b, c, d^ among amaranths = means in the same row with different superscripts differ (*P* < 0.05).

In regard to CP degradability, CS had a higher (*P* < 0.001) level of ‘a’ and ADICP, but lower (*P* < 0.001) ‘b’, ERDP, DUP, and MP, compared to the AMS mean values. The A5 silage had the highest soluble CP fraction ‘a’ (*P* < 0.001). The Maria silage had the highest (*P* < 0.001) insoluble-degradable CP fraction. The highest level of ADICP was recorded in A12 and the lowest in Maria. Maximum and minimum (*P* < 0.001) concentrations of ADICP were obtained in A12 and Maria, respectively. The A28 silage had the highest (*P* < 0.01) ERDP and MP. The DUP of A12 and Maria silages were higher (*P* < 0.001) than the other AMS.

## DISCUSSION

### Yield, Chemical Analyses, and Antinutritional Factors

Although corn forage and silage had higher DM, OM, and ME yields than the mean amaranth value, its yield was less than optimum, probably due to the late cultivation time (25^th^ of August), when there is a decline in daylight hours. Studies have shown that declining exposure to sunlight reduces crop yield by weakening the photosynthesis process (Zhang et al., 2013). Amaranth can adapt to medium temperatures (Ofitserov, 2001), but lower exposure to sunlight is unfavorable to growth and subsequent yield (Modi, 2007). The A12 cultivar appeared to be better adapted to the climate conditions in this study. However, Abbasi et al. (2012) reported higher amaranth yields (up to 16.6 t DM/ha) compared to the current study, but levels of N fertilizer were higher (240 *vs.* 53 kg urea-N/ha) and the amaranth was planted a month earlier (warmer climate) and these factors would have contributed to a higher yield. Nevertheless, this study only measured yield from one year so further research is warranted.

In many parts of the World, these forages are sown as second crops in the middle of summer after grain crops (barley or wheat) and harvested in the autumn. This leads to a low DM value (< 300 g/kg fresh weight) in both corn and amaranth because lower heat and sunlight levels in this season prevent optimum plant dry matter for silage (Nikkhah et al., 2011). A low forage DM increases the difficulty in making good quality silage (McDonald et al., 1991), which was seen when comparing AMS to CS. The A5 and A28 cultivars had the lowest DM and consequently produced the lowest quality silage (low lactic acid levels, high pH and ammonia-N). Late harvesting (last week of October) of AMS has resulted in insufficient heat and sunshine for obtaining optimum DM to enhance the ensilability characteristics (*i.e.,* most AMS were ensiled with <250 g/kg DM). Excessive moisture interferes with the rapid establishment of lactic acid-producing bacteria and delays a rapidly needed decline in pH (McDonald et al., 1991), thus contributed to lower silage quality of AMS.

High CP levels in amaranth silages (average 149 g/kg DM) and relatively low levels of NDFom (340 g/kg DM) and ADL (*i.e.,* 61.7g/kg DM, comparable to high-quality alfalfa at 60 g/kg DM, MAFF, 1992) shows its suitability as a ruminant feed, as high level of the latter compound can reduce feed intake and digestibility (Van Soest, 1994). These different levels of CP and NDFom, WSC, and NFC in amaranth compared to corn may be partially accounted for by differences in genetics, metabolism, and carbon uptake per unit area (Edwards and Walker, 1983). The silage process reduces CP levels as plant and microbial proteolysis converts these compounds into NH_3_-N (McDonald et al., 1991), and this may affect the amaranth cultivars more because of their higher moisture content. Shadi et al. (2020) reported higher NDFom in AMS (*i.e.,* 381 g/kg DM), which may be due to the growth stage at harvest (*i.e.,*Shadi et al. (2020) harvested at 93 days compared to 60 days in this study). The Maria and A12 silages had higher CP values than the other AMS which is likely a result of less dilution by ash in some varieties.

Karimi Rahjerdi et al. (2015) recorded differences in chemistry among amaranth silage cultivars. The corn forage had the optimal WSC values (60–80 g/kg DM; Wang et al. (2018) for good quality silage whereas amaranth forages had WSC values below 60 g/kg DM leading to medium silage quality. Different amounts of residual WSC among AMS was an indication of differences in silage microbe fermentation (McDonald et al., 1991). The higher EE levels in AMS in comparison with forages were caused by changes in proportions of WSC, protein, and NDF. The nature of amaranth being a halophyte means that it has higher ash levels compared to corn (Edwards and Walker, 1983). Also, wilting amaranth may also increase ash concentration through soil contamination. High ash levels in the diet from amaranth can raise concerns about dietary energy and mineral imbalance. The CS Ca:P ratio at 2:1 is recommended by NRC (2005) for the prevention of the production of urinary calculi in sheep, whereas the AMS Ca:P ratio varied between 2.5:1 and 3.0:1, so an adjustment is needed if AMS is part of the diet. This ash level and high Ca:P ratio in AMS mean that it can be used to balance dietary Ca. Amaranth ash values in this study were similar to values recorded by Shadi et al. (2020); 182 to 215 g/kg DM) but higher than values recorded by Rezaei et al. (2014) and Karimi Rahjerdi et al. (2015), at 110 and 138 g/kg DM, respectively. These differences relate to cultivar, growth stage when harvested and local soil and climate conditions (Viglasky et al., 2009).

Amaranth forage nitrate values were higher than those in corn but below values considered toxic to ruminants (*i.e.,* < 6 g/kg DM; Radostits et al., 2007). A28 forage had higher CP and lower nitrate levels than other amaranths suggesting higher levels of photosynthesis and nitrate to protein conversion (Hopkins and Hüner, 2008). Differences in nitrate levels among AMS (0.36) were smaller in comparison with their forages (1.5), as the silage fermentation process converts some nitrate to ammonia (Van Soest, 1994). Shadi et al. (2020) recorded lower nitrate values (i.e., 0.3–0.69 g/kg DM) at a 93 day harvest than in this study’s 60-day harvest suggesting a lowering of nitrate with plant maturity (Abbasi et al., 2012).

There was a difference in oxalate levels between corn and mean of the amaranths, and also among the amaranths, both in terms of forage and silage but the levels were all below 20 g/kg DM, the level considered to be toxic for ruminants (Rahman et al., 2013). Other studies have reported a wide variation of oxalic acid levels in amaranths (i.e., 2.0–114 g/kg DM; Teutonico and Knorr, 1985), relating to soil conditions, plant growth stage, and plant variety (Bressani, 1993). Bacterial degradation of oxalic acid in the silage fermentation process tends to reduce the levels (Rahman et al., 2021). Total phenolic and total tannin levels in amaranth and corn (forage and silage) were below 10 g/kg DM and had no detrimental effect (Benchaar et al., 2008) on *in vitro* fermentation. Shadi et al. (2020) reported similar levels of total phenolics (4.1–7.0 g/kg DM) and total tannins (1.7–4.9 g/kg DM). Differences in total phenolic levels among AMS (3.5) were higher than among the forages (2.9), indicating differences in silage pH, bacterial activity and extent of fermentation (Makkar and Singh, 1993).

### Silage Fermentation Characteristics

The lower pH of CS in comparison with the AMS mean pH (Table 4) relates to the higher WSC level in the corn forage (Table 2) as WSC is converted into lactic acid (Kaiser et al., 2004). The amaranths produced medium quality silage in line with their levels of DM and WSC (Faithfull, 2002). The A28 silage had the highest pH which reflected it having the lowest WSC level in the forage and the lowest lactic acid and highest ammonia-N levels in the made silage. AMS in this study had higher pH values than those recorded by Rezaei (pH = 3.9) et al. (2014), but, compare well to the mean pH (4.6) recorded by Seguin et al. (2013) of amaranth silages harvested at different growth stages. Silage pH is affected by forage WSC level, forage buffering capacity and lactic acid and ammonia-N levels accumulated in the made silage (McDonald et al., 1991; Kaiser et al., 2004). Lower levels of ammonia-N in CS in comparison with AMS are due to the lower CP level and pH resulting in less protein being degraded in the fermentation process (Kaiser et al., 2004). The AMS were low DM silages and as such had relatively low ammonia-N levels (Chamberlain and Wilkinson, 2000) indicating reasonable quality. Ammonia-N levels in the AMS were higher than that recorded by Karimi Rahjerdi et al. (2015) (40 g/kg total N), but in line with those recorded by Rezaei et al. (2014, 2015) (60.2–69.3 g/kg total N, respectively). The AMS lactic acid levels were within the 55–63 g/kg DM range accepted as adequate for medium quality silage (ZoBell et al. 2004) and in line with levels recorded by Shadi et al. (2020). Rezaei et al. (2015) reported higher lactic acid concentration (69.1 g/kg DM), relating to higher WSC values in the forage (76.9 g/kg DM) that produced lactic acid from homolactic fermentation (McDonald et al., 1991). In CS, acetic acid levels were higher than the AMS mean value which indicates more heterolactic fermentation (Kaiser et al., 2004). These higher acetic acid concentrations in CS were also reported by Rezaei et al. (2014) and Shadi et al. (2020). The concentration of butyric acid was low in all silages and is an indication of minimal clostridial fermentation (Kaiser et al., 2004). Nitrate levels are negatively correlated with clostridia numbers (Spoelstra, 1983) as during fermentation nitrate is converted into nitrite and nitric oxide which are toxic to clostridia (Weissbach, 1996).

### In vitro Gas Production and Fermentation Variables

Table 5 shows that both the CS and all the AMS have values of in vitro pH between 5.9 and 7 that are considered to be acceptable physiological levels (Wales et al. 2004), and all ammonia-N values were higher than that needed for optimum ruminal microbial growth (>50 mg/L) (Satter and Slyter, 1974). The ammonia-N levels in AMS were higher than those in CS, and CP levels were higher in AMS, likely contributing to this difference (Table 2).

The CS had higher levels of VFA and acetic acid in comparison with the mean values of the AMS which is related to its higher levels of OM (Table 2) and TDS (Table 6) leading to an increase in microbial activity (Van Soest, 1994). The Maria and A12 silages had higher VFA levels than the other cultivars and also higher OM levels. The higher cellulolytic bacteria numbers in CS would explain the higher acetic acid levels in comparison with AMS as these bacteria produce acetic acid as an endpoint of OM fermentation (Newbold et al., 2015). The similar acetic acid levels among the AMS could be correlated to their similar cellulolytic bacteria numbers (Newbold et al., 2015).

The higher protozoa numbers from CS in comparison with the mean value from AMS relate to its lower level of phenolics and the higher acetic acid level as there is a correlation between acetic acid level and protozoa population, due to the role of protozoa in the synthesis of VFA and feed degradation (Newbold et al., 2015).

Results for all silage samples identify four genera of protozoa with *Entodinium* being the dominant genus (Newbold et al., 2015).

The higher values of GP_24_, OMD, ME, and TDS from CS compared to AMS were similar to the results of Lotfi et al. (2022) and Shadi et al. (2020) and may be due to higher levels of NFC, cellulolytic bacteria and OM in CS (Van Soest, 1994). The higher mean PF_24_ value of AMS compared to CS could have been due to its higher ash level as ash does not add to GP (Makkar, 2010) The lower MCP of CS in comparison with AMS relates to its lower CP level and higher numbers of protozoa (Dehority, 2003). Of the AMS varieties, A12 had the highest in vitro GP_24_, OMD, ME, and TDS and the lowest ash level so in proportion had a higher level of fermentable substrate per unit of DM (Van Soest, 1994). Lotfi et al. (2022) and Shadi et al. (2020) reported a high GP_24_ (145 mL/g DM) from A5 as it had the highest OM level of the amaranth silages tested. The AMS, A12 had a PF_24_ in the range for conventional feedstuffs (2.75–4.41) (Makkar, 2010).

The mean value of in vitro methane produced from AMS was lower than CS and that is related to the lower protozoa numbers which are likely a result of the lower hydrogen production related to different fermentation pathways producing less hydrogen in fermentation with AMS (Newbold et al., 2015). Also, the lower acetic acid levels in AMS correlate with a lower methane output (Newbold et al., 2015). Lotfi et al. (2022) and Shadi et al. (2020) recorded similar results.

### In situ Degradability of DM and CP

In terms of DM degradability, the mean of fraction ‘aʹ from the AMS was higher in comparison with CS may relate to its higher soluble and very rapidly CP degradable fraction (g/kg DM) in AMS compared to CS (Table 7), and this may have led to higher soluble and very rapidly DM degradable fraction in AMS compared to CS. In CS, the higher fraction ‘bʹ relates to its higher level of NDFom as this is known to slowly degrade in the rumen (NRC, 2001). The higher ED of DM of CS compared to the mean AMS value relates to its higher NFC value. In terms of differences among the AMS, the lower ‘aʹ fraction of Maria and A28 in comparison with A5, A12, and A14 relates to its lower NFC levels (Table 1). Whereas, A12 had a higher ‘bʹ fraction and ED compared to the other AMS which may be due to its higher NDF level (Table 2). Another study where amaranths were grown in drier conditions recorded A5 having the highest ED of DM (632 g/kg DM) and A28 the lowest (509 g/kg DM) (Shadi et al., 2020). However, when Lotfi et al (2022) grew amaranth in hot humid conditions, the highest ED of DM was recorded for A14 (619 g/kg DM) with A5 having the lowest (593 g/kg DM). Karimi-Rahjerdi et al. (2015) recorded higher DM degradability (808–812 g/kg DM) for the two varieties, Kharkovskiy and Sem which may reflect differences in plant chemistry, climate, soil management and silage production methods (Van Soest, 1994).

In terms of the degradability of CP, the higher fractions ‘aʹ, ‘bʹ, ED, ERDP, and DUP in AMS in comparison with CS may relate to their higher CP levels as this was reported by Lotfi et al., (2022) and Shadi et al. (2020). The AMS also had a higher DUP in comparison with CS, indicating that higher amounts of CP being digested post-rumen resulted in more efficiency in CP use (Van Soest, 1994). Among AMS, A28 had higher CP quality indices (ED, ERDP, and MP), which relates to its higher CP and low ADICP. However, synchronization between energy and protein in the rumen is needed to improve ERDP utilization (NRC, 2001). Maria silage had a lower ED of CP in comparison with A28, but its MP compared well to A28 as it had the highest OM and NFC and a high level of CP. The higher DUP of Maria in comparison with the other AMS, would likely cause more efficient CP utilization as more protein was digested post-ruminally (Van Soest, 1994). Differences in DUP in AMS relate to CP levels and increases in protein breakdown in the rumen (Do et al., 2013). This means that CP degradability and silage MP are affected by different silage components and they should all be taken into account in the results (McDonald et al., 1991).

## CONCLUSIONS

The chemistry, particularly the CP and MP, and OMD of AMS suggests that this silage has potential as a feed for ruminants. The anti-nutrient levels in the tested varieties were below the toxic levels for ruminants. In terms of the forage yield, CP and MP, Maria and A28 had the highest levels but regarding OMD content, A5 and A12 had the highest levels. Overall, it can be concluded that A12 silage has the best balance of nutritive value and digestibility compared to other varieties. However, more *in vivo* study is required to confirm these nutritional characteristics.
